# Regulation of myostatin expression is associated with growth and muscle development in commercial broiler and DMC muscle

**DOI:** 10.1007/s11033-018-4187-7

**Published:** 2018-05-08

**Authors:** Tengfei Dou, Zhengtian Li, Kun Wang, Lixian Liu, Hua Rong, Zhiqiang Xu, Ying Huang, Dahai Gu, Xiaobo Chen, Wenyuan Hu, Jiarong Zhang, Sumei Zhao, Markandeya Jois, Qihua Li, Changrong Ge, Marinus F. W. te Pas, Junjing Jia

**Affiliations:** 1 Yunnan Provincial Key Laboratory of Animal Nutrition and Feed, Yunnan Agricultural University, Kunming, 650201 Yunnan Province People’s Republic of China; 2 Faculty of Food Science, Yunnan Agricultural University, Kunming, 650201 Yunnan Province People’s Republic of China; 3 Department of Agricultural Sciences, School of Life Sciences, Faculty of Science, Technology and Engineering, La Trobe University, Bundoora, VIC 3086 Australia; 4 Animal Breeding and Genomics Centre, Wageningen UR Livestock Research, Wageningen University and Research Centre, Building 107, Radix, Droevendaalsesteeg 1, 6708 PB Wageningen, The Netherlands; 5 Kunming Agricultural University, Kunming, 650201 Yunnan Province People’s Republic of China; 6 Dali University, Dali, People’s Republic of China

**Keywords:** Commercial broiler chicken, Daweishan mini chicken, Myostatin, mRNA expression, Muscle weight, Growth rate

## Abstract

Myostatin is a negative regulator of skeletal muscle growth. Muscle tissue is the largest tissue in the body and influences body growth. Commercial Avian broiler chickens are selected for high growth rate and muscularity. Daweishan mini chickens are a slow growing small-sized chicken breed. We investigated the relations between muscle (breast and leg) myostatin mRNA expression and body and muscle growth. Twenty chickens per breed were slaughtered at 0, 30, 60, 90, 120, and 150 days of age. Body and muscle weights were higher at all times in Avian chickens. Breast muscle myostatin expression was higher in Avian chickens than in Daweishan mini chickens at day 30. Myostatin expression peaked at day 60 in Daweishan mini chickens and expression remained higher in breast muscle. Daweishan mini chickens myostatin expression correlated positively with carcass weight, breast and leg muscle weight from day 0 to 60, and correlated negatively with body weight from day 90 to 150, while myostatin expression in Avian chickens was negatively correlated with carcass and muscle weight from day 90 to 150. The results suggest that myostatin expression is related to regulation of body growth and muscle development, with two different regulatory mechanisms that switch between days 30 and 60.

**Electronic supplementary material:**

The online version of this article (10.1007/s11033-018-4187-7) contains supplementary material, which is available to authorized users.

## Introduction

Muscle tissue is the largest tissue in the body and may directly influence whole body growth. Myostatin regulates muscle fibre growth [1] and muscle development via regulating satellite cell activation and renewal [2]. In broiler chicken myostatin haplotypes were reported to be associated with body weight [3]. The relationship between myostatin mRNA expression, growth rate, muscle mass, and body weight in chickens is poorly understood. We used commercial Avian broiler (AB) and Daweishan mini chickens (DMC) as a model system to investigate these relationships. Broiler chickens are characterized by a high body weight at slaughter and extremely fast growth rate from hatching to slaughter [4, 5], while DMC are a slow growing low body weight breed [6]. The combination of these two breeds provides a perfect model system to investigate the biological mechanisms underlying growth rate and body weight in chickens. The objective of this research was to investigate the relationship between myostatin expression in breast and leg muscle and whole body growth rate, muscle development, and growth rate.

## Materials and methods

### Animals and experiments

This study was approved by the Animal care and use committee of the Yunnan Province of P. R. China and all the experiments complied with the requirements of the directory of the Ethical Treatment of Experimental Animals of China. DMC were purchased from the Chicken Farm of Yunnan Agricultural University within the first day of life. Commercial AB were purchased from the Chicken Farm (Kunming Zhengda Group, a source from the American IVY International Co., LTD). This study used 120 chickens per breed. At each time point (0, 30, 60, 90, 120, and 150 days of age) 20 animals per breed were sacrificed. The chicken were reared under standard conditions on starter diets to day 30, and then on adult chicken diets to day 150. Table 1 shows the diet compositions and some egg characteristics of the two chicken breeds.

**Table 1:** Compositions and nutrient levels of the chicken diets, and egg characteristics of the two chicken breeds

	Avian broiler chicken	Daweishan mini chicken
Age	40 weeks-old	30 weeks-old
Nutrition		
Metabolizable energy (Kcal kg^–1^)	2760	2750
Crude protein (%)	16.0	15.6
Calcium (%)	3.07	3.00
Total phosphorus (%)	0.66	0.60
Available phosphorus (%)	0.37	0.38
Salt (%)	0.37	0.37
Lys (%)	0.82	0.76
Met (%)	0.42	0.35
Methionine + cystinol (%)	0.70	0.63
Egg weight (mean ± SD)	57.63 ± 3.44	35.52 ± 4.10
Egg shape index (mean ± SD)	1.37 ± 0.07	1.30 ± 0.04
Chick birth weight (Mean ± SD)	48.19 ± 4.02	20.70 ± 2.50

Chickens received the starter diet from day 0 to 30. From day 30 to 150 the chickens received the adult chicken diet

The chickens had free access to feed and water during the entire rearing period. The chickens were reared in an environmentally controlled room. The brooding temperature was maintained at 35 °C for the first 2 days, and then decreased gradually to 22 °C until 30 days. At 30 days of age, the chickens were randomly allocated to individual metabolism cages in an enclosed room, with ambient temperatures varying from 21 to 24 °C on a light:dark cycle of 12:12 h.

### Measurement of growth performance and carcass traits

Chickens were weighed on a tarred digital scale (Shanghai Yizhan weighing apparatus limited company, YZ 0.01–10 kg, China). Body weights were determined in the morning following a 16 h fasting. The body weights were determined until week 20 because the DMC reached sexual maturity at approximately 20 weeks. The carcass weight was measured after the blood and feathers had been removed. After removal of the oesophagus, trachea, gastrointestinal tract, pancreas, spleen, and gonads the semi-eviscerated weight was measured, and after removal of the head, heart, claws, liver, glandular stomach, gizzard, and abdominal fat the eviscerated weight was measured. The dressing percentage was calculated by dividing the carcass weight by the body weight. The percentage of carcass weight originating from of each carcass trait (eviscerated weight, semi-eviscerated weight, breast muscle weight, leg muscle weight, and abdominal fat weight) was calculated by dividing each trait by the carcass weight [7].

### Myostatin real-time PCR

Real-time PCR was performed to quantify skeletal muscle myostatin mRNA expression as described previously [8]. Breast and leg muscle samples were collected within 20 min after chickens were sacrificed. Samples were immediately placed in sterile tubes (RNase-free), snap frozen in liquid nitrogen, and stored at − 80 °C. Total RNA was isolated using Trizol-Reagent (Invitrogen, USA) and reverse transcribed using oligo (dT) 12–18, random primers, and M-MLV reverse transcriptase (Invitrogen, USA).

Real-time PCR analysis was performed using the iCycler Real Time Detection System (Bio-Rad Laboratories, Inc. USA) and SYBR Green master mix (iQTM SYBR-Green® Supermix, Dalian TaKaRa Biotechnology Co. Ltd. Add). The myostatin primers used were 5′-GCTTTTGATGAGACTGGACGAG-3′ (forward) and 5′-AGCGGGTAGCGACAACATC-3′ (reverse), and the annealing temperature was 60 °C. The 18S rRNA gene was used as a reference: 5′CGCGTGCATTTATCAGACCA-3′ (forward) and 5′-ACCCGTGGTCACCATGGTA-3′ (reverse), annealing temperature 58 °C. Primers were commercially synthesized (Shanghai Shenggong Biochemistry Company P.R.C). The PCR reactions were performed in 25 µl volumes containing 12.5 µl of iQ™ SYBR Green Supermix, 0.5 µl (10 mmol l^−1^) of each primer, and 1 µl of cDNA. Amplification and detection of products was performed with the following cycle profile: one cycle of 95 °C for 2 min, and 40 cycles of 95 °C for 15 s, annealing temperature for 30 s, and 72 °C for 30 s, followed by a final cycle of 72 °C for 10 min. The specificity of the amplification product was verified by electrophoresis on a 0.8% agarose gel and DNA sequencing.

### Statistical analysis

Carcass trait data were expressed as the mean ± SE for the two breeds. SAS version 9.3 was used for all analyses as described by Arounleut et al. [9]. Myostatin mRNA expression levels were expressed relative to 18S. Differences in temporal gene expression and comparisons between breast and leg muscle were analyzed using a *T* test. Both breeds were tested individually across all time points (0–150 day of age). Correlation analyses between myostatin expression and carcass traits were performed using the Spearman correlation procedure, which was chosen because of the nonparametric nature of the data. The CORR procedure of SAS (SAS version 9.3) was used to perform the correlations. Significance and residual values were calculated using a two-way ANOVA. *P* < 0.05 was used to determine statistical significance.

## Results

### The model system

**Fig. 1:**
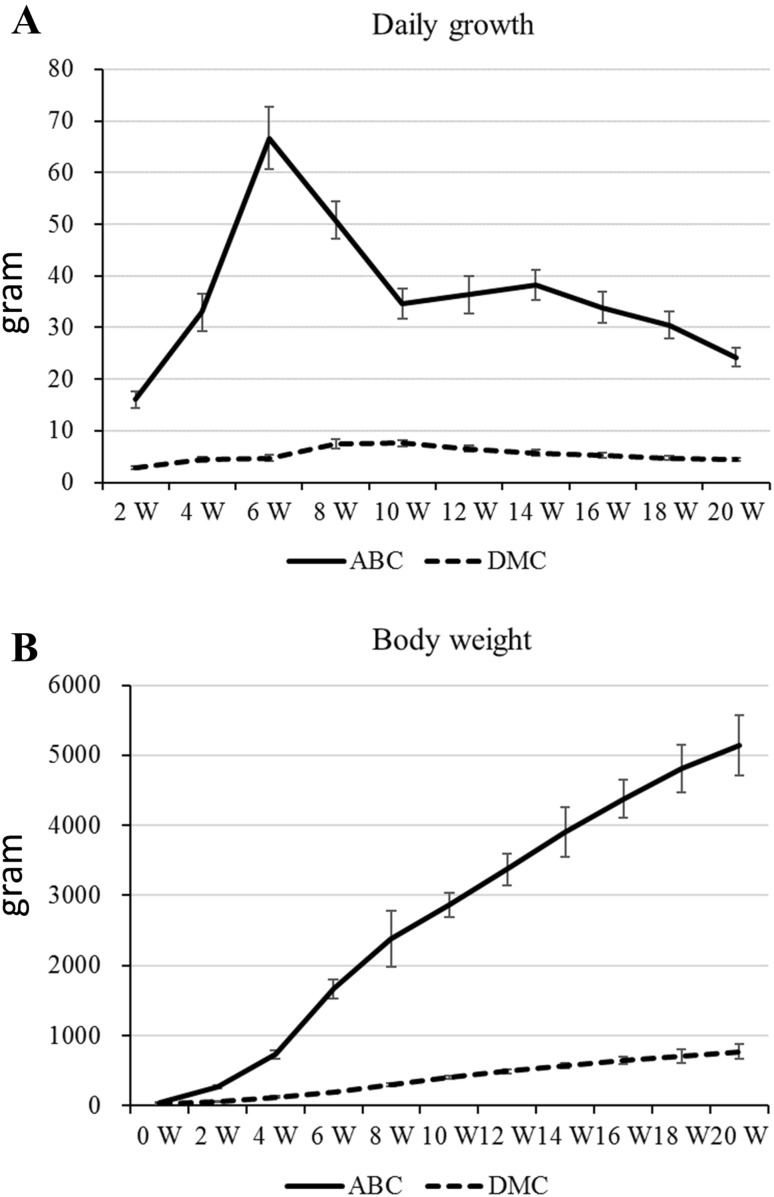
Daily growth rate (**a**) and body weight (**b**) of the ABC and the DMC. Data are in grams

Figure 1 shows the growth rate (Fig. 1a) and body weight (Fig. 1b) data for the two chickens. At all ages the AB line chickens showed a higher growth rate than the DMC chickens. The commercial AB line chickens showed a high increased daily growth rate from hatching to 5 weeks of age after which the growth rate decreased. The DMC chickens showed a small increase in average daily growth rate until week 8 followed by a small decline of the average daily growth. The DMC chickens showed no peak like the AB line chickens for growth rate. As a consequence of this the body weight of the AB line chickens increased more sharply than the DMC and reached a much higher body weight at 20 weeks (Fig. 1b).

**Fig. 2:**
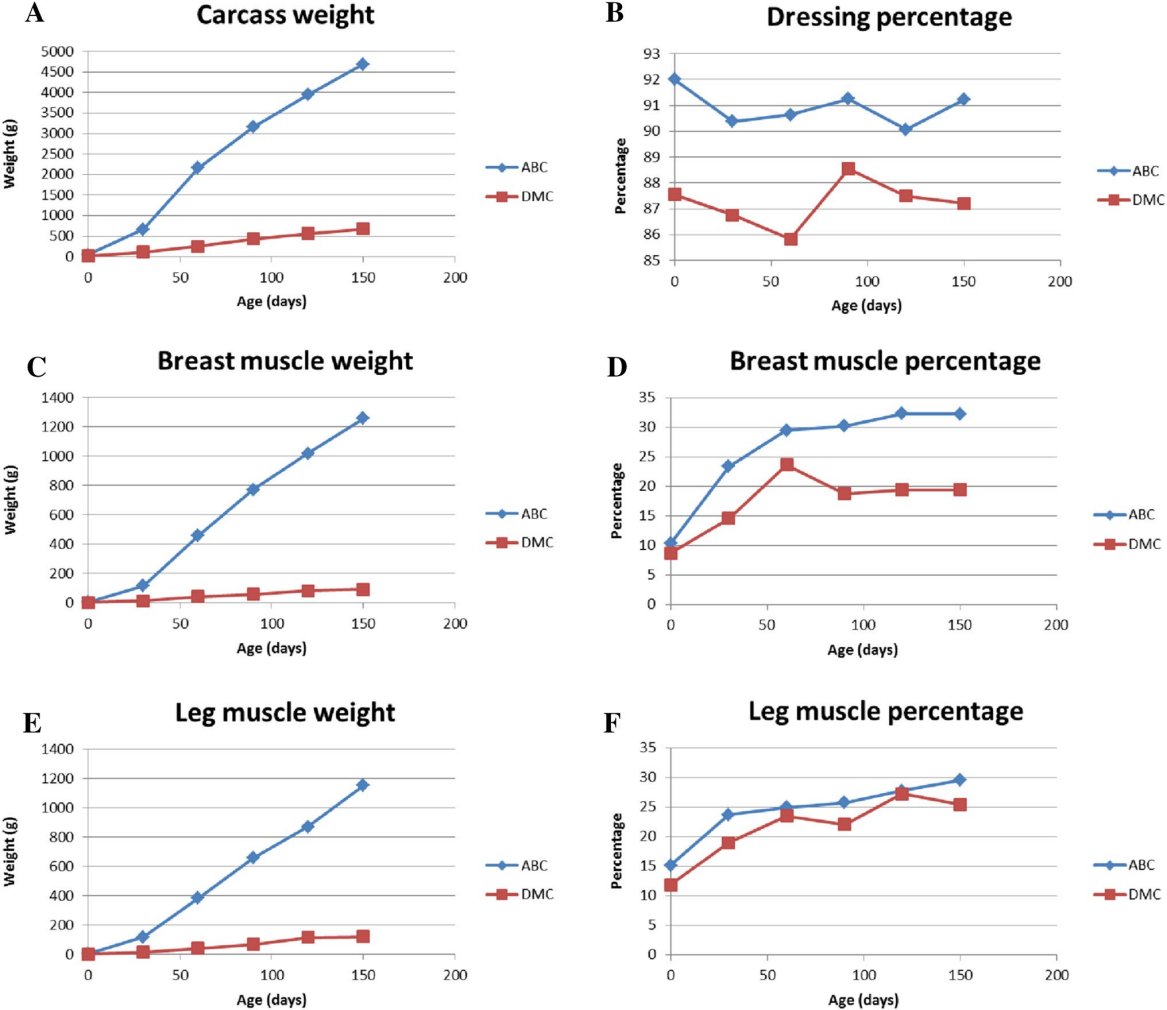
Developmental changes in commercial AB (ABC) and DMC (DMC) from birth to 150 days of age. **a, c, e** Measured weights (g) of carcass, breast muscle, and leg muscle, respectively; **b, d, f** calculated percentages of dressing, breast muscle, and leg muscle, respectively. The dressing percentage was the carcass weight divided by the body weight. The muscle percentages were calculated by dividing muscle weight by carcass weight. All measurements differed between ABC and DMC at all time points except for breast muscle percentage (**d**) at birth (0 days) and leg muscle percentage at day 0, 30, 60, and 120

Figure 2 shows the carcass weight gain and muscle development. The carcass weights (Fig. 2a) of the DMC were significantly lower than the commercial AB during the entire growth period. The carcass weight, breast muscle weight, and leg muscle weight (Fig. 2a, c, e) increased faster after day 30 than during the first 30 days of life. The dressing percentage (Fig. 2b), which is the amount of meat on the carcass, indicates that muscle content was higher in the AB carcasses. Both breast (Fig. 2c) and leg muscle (Fig. 2e) growth rates were closely related to carcass weight. Higher breast muscle percentage (Fig. 2d), but not leg muscle percentage (Fig. 2f) was observed in the AB compared to the DMC. These observations were confirmed by additional carcass measurements (Table 2, Supplementary material 1).

**Table 2:** Carcass measurements of the Avian broiler line and the DMC breed at all sampled ages

Carcass traits	Age					
	0 days	30 days	60 days	90 days	120 days	150 days
AB						
Carcass weight (g)^a^	44.38	662.85	2156.57	3158.65	3941.50	4690.67
Semi-eviscerated weight (g)^b^	39.81	606.35	1762.16	2839.19	3557.87	4283.46
Eviscerated weight (g)^c^	34.04	494.04	1541.42	2549.19	3129.87	3884.63
Breast muscle weight (g)	3.56	116.08	457.55	770.17	1017.50	1257.67
Leg muscle weight (g)	5.14	117.54	384.47	657.23	869.67	1151.62
Dressing percentage (%)^d^	92.01	90.38	90.63	91.25	90.07	91.22
Semi-eviscerated percentage (%)^e^	82.50	82.65	81.72	82.14	81.42	83.31
Eviscerated percentage (%)^e^	70.53	74.49	71.52	73.77	71.55	75.58
Breast muscle percentage (%)^e^	10.41	23.39	29.54	30.23	32.38	32.29
Leg muscle percentage (%)^e^	15.18	23.71	24.99	25.79	27.80	29.58
Abdominal fat percentage (%)^e^				4.07	5.56	5.33
Abdominal fat weight (g)^e^				101.24	167.33	196.00
DMC						
Carcass weight (g)^a^	18.12	107.23	251.3	434.29	564.28	673.23
Semi-eviscerated weight (g)^b^	15.6	88.84	214.5	365.51	502.28	577.31
Eviscerated weight (g)^c^	12.96	79.19	180.83	306.1	415.86	477.59
Breast muscle weight (g)	1.13	11.55	41.16	57.77	80.9	92.53
Leg muscle weight (g)	1.52	15.05	42.43	67.83	114.14	121.46
Dressing percentage (%)^d^	87.55	86.76	85.83	88.54	87.49	87.2
Semi-eviscerated percentage (%)^e^	75.14	71.89	73.23	74.67	77.88	74.84
Eviscerated percentage (%)^e^	62.45	64.09	61.86	62.58	64.41	61.81
Breast muscle percentage (%)^e^	8.71	14.62	23.67	18.8	19.43	19.45
Leg muscle percentage (%)^e^	11.88	18.98	23.55	22.12	27.28	25.47
Abdominal fat percentage (%)^e^				3.14	5.08	4.51
Abdominal fat weight (g)^e^				9.52	20.43	20.31

Carcass measurements were done after subsequently dissection of various organs as detailed in the footnotes

^a^The carcass weight was measured after the blood and feathers had been dissected

^b^The semi-eviscerated weight was measured after subsequent dissection of the oesophagus, trachea, gastrointestinal tract, pancreas, spleen, and gonads

^c^the eviscerated weight was measured after subsequent dissection of the head, heart, claws, liver, glandular stomach, gizzard and abdominal fat

^d^The dressing percentage was determined as the proportion of the carcass weight of the body weight

^e^The proportions of the weights from each of the carcass traits (eviscerated weight, semi-eviscerated weight, breast muscle weight, leg muscle weight, and abdominal fat weight) of the carcass weight were calculated as eviscerated percentage, semi-eviscerated percentage, breast muscle percentage, leg muscle percentage, and abdominal fat percentage, respectively

### Myostatin mRNA expression

**Fig. 3:**
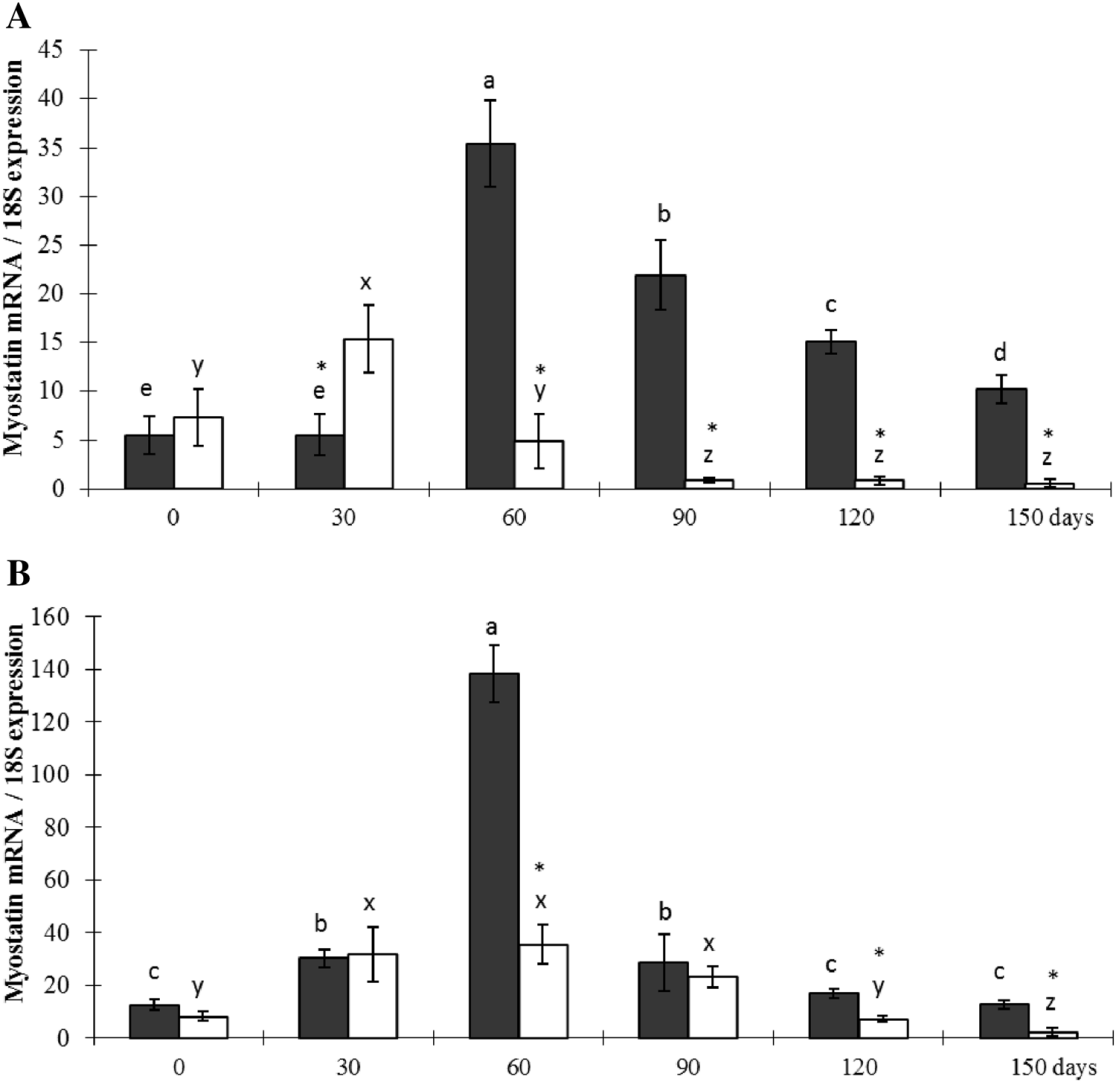
Myostatin expression of the breast muscle of DMC (BM-DMC) and commercial AB (BM-ABC), and the leg muscle of DMC (LM-DMC) and commercial AB (LM-ABC) at 0, 30, 60, 90, 120, and 150 days of age. Data are expressed as the mean ratio ± SE of specific myostatin mRNA:18S rRNA for each time point from the two breeds. Myostatin expression differences (P < 0.05) were evaluated between time points within each breed (lower case letters) and between the two breeds for each time point (*)

Figure 3 shows myostatin expression levels of breast and leg muscle. Myostatin expression in leg muscle was higher than in breast muscle in the AB, with the largest difference observed on days 60–120. The smallest difference was observed on days 0 and 30. Differences between breast and leg muscle myostatin expression in the DMC were only observed at day 60.

Breast muscle myostatin expression was higher in the AB than the DMC at day 30. In contrast, breast and leg muscle myostatin expression was higher in DMC than AB from day 60 to day 150. Myostatin expression in DMC reached a peak level at day 60 in both muscles, which was not observed in the AB. Although breast muscle myostatin expression decreased after day 60 in the DMC, it remained relatively high compared to AB. Leg muscle myostatin expression also decreased after day 60 in the DMC, reaching the same overall levels observed in the AB on days 90 and 120. There remained a small but significant difference in myostatin expression between the two breeds at days 120 and 150.

### Correlation between animal traits and myostatin expression

Figure 3 shows differences in myostatin expression between day 0 and day 30, and from day 60 onwards. This indicates that important changes in the regulation and effect of myostatin may occur between 30 and 60 days of age. Therefore, we correlated myostatin expression with the measured production traits for time points ranging from day 0 to 30, day 60 to 150 (Table 3), day 0 to 60, and day 90 to 150 (Table 4). The results differ for days 30 to 60 as the end of period 1, the period where the largest differences in myostatin expression were found (Fig. 3). The analyses showed high similarity between the breeds for the correlations. The correlations for the commercial AB were significant for day 0–30 and day 60–150, but not day 0–60 and day 90–150. A reverse in the direction of the correlation was observed for the DMC. This may indicate that myostatin expression is differently regulated between the two chicken breeds.

**Table 3:** Correlations between myostatin expression and developmental traits in the commercial AB and DMC for day 0–30 and day 60–150

	AB						DMC					
	Breast muscle			Leg muscle			Breast muscle			Leg muscle		
	Correlation	Residual	P-value	Correlation	Residual	P-value	Correlation	Residual	P-value	Correlation	Residual	P-value
0–30 days												
Carcass weight	0.672	824.7	< 0.001	0.73	4965.4	< 0.001	0.037	535.4	0.822	0.403	12604.5	0.01
Breast muscle weight	0.676	817.9	< 0.001	0.7	5416.9	< 0.001	0.05	534.8	0.76	0.395	12697.2	0.012
Leg muscle weight	0.664	841.5	< 0.001	0.7	5420.6	< 0.001	0.047	534.9	0.773	0.41	12521.1	0.009
Breast muscle myostatin expression	1			0.59	981.9	< 0.001	1			− 0.522	390.0	0.001
Leg muscle myostatin expression	0.59	197.2	< 0.001	1			− 0.522	0.001	390.066	1		
60–150 days												
Carcass weight	− 0.548	347.8	< 0.001	− 0.634	16881.9	< 0.001	− 0.296	40790.6	0.008	− 0.536	275410.9	< 0.001
Breast muscle weight	− 0.526	359.6	< 0.001	− 0.623	17286.7	< 0.001	− 0.154	43639.7	0.174	− 0.351	339020.9	< 0.001
Leg muscle weight	− 0.505	370.7	< 0.001	− 0.597	18190.9	< 0.001	− 0.192	43051.9	0.089	− 0.394	326746.3	< 0.001
Breast muscle myostatin expression	1			0.216	474.2	< 0.001	1			0.349	39292.1	0.002
Leg muscle myostatin expression	0.216	474.2	0.055	1			0.349	38358.0	0.001	1		

Myostatin expression peaked at day 60, which may have been initiated between day 30 and 60. Therefore, the correlations were calculated from day 0 to 30, and from day 60 to 150. Correlation analyses were performed using the Spearman correlation procedure with the CORR procedure of SAS (SAS version 9.3). Significance and residual values were calculated using a two-way ANOVA

**Table 4:** Correlations between myostatin expression and developmental traits in commercial AB and DMC for day 0 to 60 and day 90 to 150

	AB						DMC					
	Breast muscle			Leg muscle			Breast muscle			Leg muscle		
	Correlation	Residual	P-value	Correlation	Residual	P-value	Correlation	Residual	P-value	Correlation	Residual	P-value
0–60 days												
Carcass weight	− 0.328	2049.6	0.01	0.445	20019.6	< 0.001	0.43	37155.8	0.001	0.671	198676.6	< 0.001
Breast muscle weight	− 0.346	2023.1	0.007	0.384	21280.7	0.002	0.467	35638.9	< 0.001	0.662	202775.3	< 0.001
Leg muscle weight	− 0.329	2049.2	0.01	0.423	20503.8	0.001	0.447	36488.4	< 0.001	0.623	220884.6	< 0.001
Breast muscle myostatin expression	1			0.221	23746.9	0.09	1			0.419	297849.5	0.001
Leg muscle myostatin expression	0.221	2185.3	0.09	1			0.419	37598.5	0.001	1		
90–150 days												
Carcass weight	− 0.43	5.2	0.001	− 0.643	5152.7	< 0.001	− 0.592	3202.1	< 0.001	− 0.324	9214.9	0.012
Breast muscle weight	− 0.439	5.2	< 0.001	− 0.601	5613.9	< 0.001	− 0.179	4774.0	0.171	− 0.018	10293.3	0.892
Leg muscle weight	− 0.416	5.3	0.001	− 0.555	6076.0	< 0.001	− 0.283	4538.3	0.029	− 0.129	10125.8	0.327
Breast muscle myostatin expression	1			0.305	7967.5	0.018	1			0.344	9081.3	0.007
Leg muscle myostatin expression	0.305	5.8	0.018	1			0.344	4350.1	0.007	1		

Myostatin expression peaked at day 60. Correlation analyses were performed using the Spearman correlation procedure with the CORR procedure of SAS (SAS version 9.3). Significance and residual values were calculated using a two-way ANOVA

Additional correlations between the muscle/meat characteristics were also calculated (Table 5, day 0–60 and day 90–150). The correlations were as expected for carcass quality data and did not differ between the two analyses differing for either day 60 or 90 separating the two developmental periods; high correlations were found between body weight and carcass traits measured after slaughter.

**Table 5:** Correlation analysis between carcass measurements of commercial AB and DMC

	Body weight			Carcass weight			Semi-eviscerated weight			Eviscerated weight			Breast muscle weight			Leg muscle weight
	Correlation	Residual	P-value	Correlation	Residual	P-value	Correlation	Residual	P-value	Correlation	Residual	P-value	Correlation	Residual	P-value	Correlation
0–60 days																
AB																
Body weight	1															
Carcass weight	0.999	93487.8	< 0.001	1												
Semi-eviscerated weight	0.995	611001.9	< 0.001	0.996	419861.1	< 0.001	1									
Eviscerated weight	0.995	636341.7	< 0.001	0.996	406052.6	< 0.001	0.997	225150.5	< 0.001	1						
Breast muscle weight	0.981	2238087.2	< 0.001	0.985	1471710.5	< 0.001	0.982	1257852.8	< 0.001	0.983	919331.6	< 0.001	1			
Leg muscle weight	0.989	1324452.8	< 0.001	0.989	1077723.8	< 0.001	0.987	903235.0	< 0.001	0.994	319429.0	< 0.001	0.977	128474.2	< 0.001	1
DMC																
Body weight	1															
Carcass weight	0.998	3161.2	< 0.001	1												
Semi-eviscerated weight	0.997	4703.6	< 0.001	0.997	4183.7	< 0.001	1									
Eviscerated weight	0.997	4125.1	< 0.001	0.997	3421.5	< 0.001	0.995	4355.5	< 0.001	1						
Breast muscle weight	0.893	164323.4	< 0.001	0.881	134157.6	< 0.001	0.888	93291.0	< 0.001	0.893	62605.9	< 0.001	1			
Leg muscle weight	0.945	86510.2	< 0.001	0.94	70405.0	< 0.001	0.938	52989.7	< 0.001	0.949	30504.9	< 0.001	0.921	2991.0	< 0.001	1
90–150 days																
AB																
Body weight	1															
Carcass weight	0.991	466670.8	< 0.001	1												
Semi-eviscerated weight	0.988	612623.0	< 0.001	0.997	143741.1	< 0.001	1									
Eviscerated weight	0.988	1322025.9	< 0.001	0.984	781546.8	< 0.001	0.987	564859.6	< 0.001	1						
Breast muscle weight	0.917	4156310.0	< 0.001	0.94	2868995.0	< 0.001	0.944	2401505.5	< 0.001	0.945	1878601.3	< 0.001	1			
Leg muscle weight	0.923	3886872.6	< 0.001	0.941	2808227.6	< 0.001	0.939	2601182.4	< 0.001	0.94	2012061.6	< 0.001	0.89	510776.8	< 0.001	1
DMC																
Body weight	1															
Carcass weight	0.992	26549.4	< 0.001	1												
Semi-eviscerated weight	0.958	137934.2	< 0.001	0.974	65259.0	< 0.001	1									
Eviscerated weight	0.947	169994.7	< 0.001	0.968	80655.0	< 0.001	0.989	24397.1	< 0.001	1						
Breast muscle weight	0.499	1246534.3	< 0.001	0.563	869831.2	< 0.001	0.623	687995.5	< 0.001	0.651	462184.5	< 0.001	1			
Leg muscle weight	0.609	1043994.2	< 0.001	0.659	721054.9	< 0.001	0.749	492872.4	< 0.001	0.768	329056.4	< 0.001	0.941	2893.9	< 0.001	1

Myostatin expression peaked at day 60. Therefore, correlations were calculated from day 0 to 60, and after day 60 to 150. Correlation analyses were performed using the Spearman correlation procedure with the CORR procedure of SAS (SAS version 9.3). Significance and residual values were calculated using a two-way ANOVA

*BW* body weight, *CW* carcass weight (= BW after removal of blood and feathers), *SEW* semi-eviscerated weight (= CW after removal of the esophagus, trachea, gastrointestinal tract, pancreas, spleen, and gonads), *EW* eviscerated weight (= SEW after removal of the head, heart, claws, liver, glandular stomach, gizzard and abdominal fat), *BMW* breast muscle weight, *LMW* leg muscle weight

## Discussion

Commercial broiler chickens have been subjected to strong human-driven selection leading to remarkable phenotypic changes in morphology, such as increased muscle growth and physiology leading to a more than 300% increase in body growth rates [10–12], accompanied by significant increases in metabolic rates [7, 13] and higher incidence of sudden death syndrome [14], associated with smaller organs. The internal organs of commercial AB develop slower, remain smaller in size, and have limited oxygen supply compared to the Daweishan chickens [15]. We observed a high correlation between body weight and carcass traits, indicating balanced growth in both breeds. The lower correlation comparing AB and DMC for the eviscerated weight from day 90 to 150 revealed higher internal organ content in the DMC.

DMC displayed low body and muscle growth rates, leading to a lower body weight compared to the commercial AB. Identifying the genetic changes underlying these developmental differences would provide new insight into the biological mechanisms by which genetic variation shapes phenotypic diversity [16]. Our results provide evidence for major differences in body growth rates and muscular development between the two breeds. The commercial AB are characterized by an extremely high dressing percentage (> 90%) due to high muscularity (> 60% for breast and leg muscles only), both of which were more moderate in the DMC (muscle content 45% for these two muscles). We conclude that comparing these two breeds together provides a large phenotypic contrast, providing an excellent model system to investigate the molecular biological mechanisms underlying the physiology of selection pressures applied to commercial AB. The lower dressing percentage of DMC compared to commercial AB observed here indicates that DMC have relatively higher internal organ weights. These differences may indicate breeding effects due to selection for high growth rate and muscle content in the commercial AB.

Breast and leg muscle myostatin expression in DMC peaked at day 60. This suggests that this developmental time point represents a crucial regulatory stage. Breast muscle myostatin expression at day 30 is higher in commercial AB than DMC, suggesting reduced muscle development in the commercial AB. Indeed a delay in the development of both muscles—especially breast muscle—and body growth rates was associated with increased myostatin expression at day 30 in the commercial AB. Nevertheless, the growth rate of commercial AB is already much higher than DMC, which may be related to the selection background of the breed.

From day 60 onwards, breast and leg muscle myostatin expression was higher in the DMC than the commercial AB. Myostatin reduces muscle growth [1] and body/carcass growth rates [17], and the body/carcass growth and muscle development rates of commercial AB is much higher than DMC. This suggests that from day 60 onwards, myostatin is a regulator of both muscle and whole body growth.

The higher myostatin expression observed in the leg muscle compared to breast muscle suggests leg muscle growth rates are affected more by myostatin than breast muscle growth rates. Therefore, we hypothesize that the regulation of myostatin expression is part of the biological mechanism underlying the response to selection for increased breast muscle development and body growth rates in commercial AB. This is in agreement with Guernec et al. [18].

Our correlation analyses between myostatin expression and body and muscle growth suggest an important switch in regulatory mechanisms between 30 and 60 days of age. The observed correlations were higher in the time period ranging between day 0 to 30 and day 0 to 60 for the commercial AB, and to a lesser extent in the time period ranging from day 60 to 150 than day 90 to 150. The opposite was observed for the DMC. We concluded that (1) the switch in regulation of myostatin expression occurs between 30 and 60 days of age in both breeds, and (2) the myostatin effect is higher in the young commercial AB than the young DMC, while the reverse conclusion can be reached for the older chickens. Myostatin expression is highly regulated via different mechanisms. These mechanisms may induce the regulatory switch. The selection pressure in the commercial AB line may have affected these regulatory mechanisms, and as a consequence the mechanisms differ between the two chicken breeds. An alternative explanation of the observed myostatin effects may be that the regulation of muscle weights and body weights differs in the two breeds. In the time period ranging from day 90 to 150, myostatin expression correlated negatively with body growth and leg muscle development in the commercial AB, and with body growth in the DMC. Higher myostatin expression was associated with reduced body growth rates and muscle development. This suggests a direct biological effect of myostatin expression on these traits. The observed lower myostatin expression, especially in the breast muscle of the commercial AB compared to the DMC could result from selection for increased breast muscle weight and whole body growth rates. Because myostatin reduces the development and size of muscle tissue, lower myostatin expression could explain the biological regulatory mechanism by which through which selection results in increased body growth rate and muscle size.

While correlation was observed between myostatin expression and body and muscle growth in young animals (0–60 days), there was a lack of association between the myostatin expression between leg and breast muscle. This again indicates that myostatin expression is differently regulated between leg and breast muscle. This may indicate that the switch in regulation of myostatin expression between 30 and 60 days of age in both breeds has finished and therefore regulatory mechanisms differ.

The situation in young animals (0–60 days) is different. No correlation between myostatin expression and body and muscle growth was observed in the young AB, suggesting myostatin expression is not involved in regulating growth rates in the high growth and muscularity broiler breed during early development.

In conclusion, our data suggest that regulation of myostatin expression may be part of a biological mechanism underlying selection for high growth rate, high muscularity, and high body weight in commercial AB. The biological effects differ before and after 60 days of age, at which point myostatin expression peaks in the slow growing chicken line. Furthermore, the biological mechanisms underlying the response to selection in breast and leg muscle seem to be different in the two growth periods. This suggests that regulation of myostatin represents only a part of the biological mechanisms at play.

## Electronic supplementary material

Below is the link to the electronic supplementary material.

Supplementary material 1. Raw data of carcass measurements and RT-PCR myostatin mRNA expression. (XLSX 32 KB)
